# 

**Published:** 1921-05-07

**Authors:** 


May 7, 1921] [Price Twopence

### The Hospital

The Workers' Newspaper of Administrative Medicine and Institutional Life, Administration, National Insurance and Health.

CONTENTS

A Hospital Pageant? ... ... ... ... ... 93

The King's Fund and Income Tax ... ... ... 94
Is a Workable Scheme Still to be Found?

Annual Meetings of Hospitals ... ... ... ... 94

Notes of the Week ... ... ... ... 95
The Prince among the Children—Changes at St. Bartholomew's—Dr. Addison at the Federation's Dinner—Tuberculosis Regulations, 1921—The Dreadnought Hospital—Dr. Dill's Scheme in London—Tobacco and Health—Remarkable Progress at Leeds—A "Crisis Passed" at Nottingham—Help from the Unemployed—A Matron's Flat—The Hospital of Lambeth.

The Health of a Child ... ... ... ... ... 97

Customs to be Condemned.

Research Defence... ... ... ... ... 98

Why Animal Experiment is Necessary.

Some International Health Questions and the League of Nations ... ... ... ... 99
An Address to the Kensington Branch of the League of Nations Union.

The Public Well-Being ... ... ... ... ... 101

Brightening Medical Reports—Industrial Health—A "Red Cross" Warning.

The Matrons' and Sisters' Department ... ... 103
The New Spirit of Reciprocity.
Freedom of the General Nursing Council Imperilled.

Round the Hospitals ... ... ... ... ... 104

The College of Nursing (Limited by Guarantee) 105
What the Local Centres are Doing.

Lectures to Creche Workers ... ... ... ... 105

The Editor's Letter-Box .. ... .. ... ... 106
The New Secretary of Westminster Hospital—The College Council Elections.

Parliament and the Professions ... ... ... 106

Nursing Authorities on Training and Teaching ... vi
The Informal Conference.

The Nurses' Missionary League ... ... ... viii

